# Daily rhythms of both host and parasite affect antimalarial drug efficacy

**DOI:** 10.1093/emph/eoab013

**Published:** 2021-04-26

**Authors:** Alíz T Y Owolabi, Sarah E Reece, Petra Schneider

**Affiliations:** Institute of Evolutionary Biology & Institute of Immunology and Infection Research, School of Biological Sciences, University of Edinburgh, Charlotte Auerbach Road, Edinburgh EH9 3FL, UK

**Keywords:** chronotherapy, malaria, *Plasmodium*, artemisinin, circadian, drug sensitivity

## Abstract

**Background and objectives:**

Circadian rhythms contribute to treatment efficacy in several non-communicable diseases. However, chronotherapy (administering drugs at a particular time-of-day) against infectious diseases has been overlooked. Yet, the daily rhythms of both hosts and disease-causing agents can impact the efficacy of drug treatment. We use the rodent malaria parasite *Plasmodium chabaudi*, to test whether the daily rhythms of hosts, parasites and their interactions affect sensitivity to the key antimalarial, artemisinin.

**Methodology:**

Asexual malaria parasites develop rhythmically in the host’s blood, in a manner timed to coordinate with host daily rhythms. Our experiments coupled or decoupled the timing of parasite and host rhythms, and we administered artemisinin at different times of day to coincide with when parasites were either at an early (ring) or later (trophozoite) developmental stage. We quantified the impacts of parasite developmental stage, and alignment of parasite and host rhythms, on drug sensitivity.

**Results:**

We find that rings were less sensitive to artemisinin than trophozoites, and this difference was exacerbated when parasite and host rhythms were misaligned, with little direct contribution of host time-of-day on its own. Furthermore, the blood concentration of haem at the point of treatment correlated positively with artemisinin efficacy but only when parasite and host rhythms were aligned.

**Conclusions and implications:**

Parasite rhythms influence drug sensitivity *in vivo*. The hitherto unknown modulation by alignment between parasite and host daily rhythms suggests that disrupting the timing of parasite development could be a novel chronotherapeutic approach.

**Lay Summary:**

We reveal that chronotherapy (providing medicines at a particular time-of-day) could improve treatment for malaria infections. Specifically, parasites’ developmental stage at the time of treatment and the coordination of timing between parasite and host both affect how well antimalarial drug treatment works.

## INTRODUCTION

That circadian rhythms affect drug treatment outcomes (including the alleviation of symptoms and/or severity of side effects) has been known for decades. Chronotherapeutic approaches aim to optimise the timing of drug administration by considering how daily changes in gene expression and physiology influence the absorption, metabolism, toxicity and half-life of drugs, the availability of drug targets, and co-active immune effectors [1]. For example, lipid-lowering medications are taken at night to coincide with the daily peak in cholesterol biosynthesis, rheumatoid arthritis is treated at night to minimise morning symptoms, and daily rhythms may affect the efficacy and toxicity of some cancer drugs (reviewed in [2]). With most best-selling human medicines targeting products of genes with daily rhythmicity, chronotherapy could optimise the use of many drugs [3]. Chronotherapy is mostly used against non-communicable diseases. Yet, timing drug treatment according to rhythms exhibited by hosts and/or parasites [4–6] could optimise drug bioavailability and half-life, minimise drug toxicity and coordinate with immune rhythms to enhance parasite removal [7–9].

Parasite rhythms are suspected to affect drug sensitivity during malaria infections. Most species of malaria (*Plasmodium*) parasites replicate synchronously in the host’s blood, with the duration of the intra-erythrocytic developmental cycle (IDC) taking multiples of 24 h depending on the species (reviewed in [10]). The IDC comprises sequential morphologically, transcriptionally and metabolically divergent developmental stages, and multiple antimalarials show stage-specific efficacy (e.g. [11, 12]). Determining which IDC stage is most vulnerable to treatment is especially useful for drugs with short half-lives [13], such as artemisinin derivatives [14, 15], which are currently used in combination therapy as first-line treatment for malaria. There is considerable variation in stage-specific sensitivity to artemisinin across studies (Table 1). Conflicting results may be due to variation between experimental set-ups, including differences in artemisinin derivatives, parasite species and genotypes, and the ages of IDC stages tested (e.g. young or old rings). Furthermore, *in vitro* and *in vivo* studies may yield different outcomes because host rhythms are likely to directly, and/or via interactions with parasite rhythms, affect drug efficacy. However, *in vivo* studies often confound IDC stage and host time-of-day at the point of treatment. For example, if a treatment seems most efficacious at dawn, it is unclear whether this is due to the effects of host time-of-day on drug metabolism, and/or corresponds to parasites being at a particularly vulnerable IDC stage. As parasite and host rhythms may not always align, knowing whether chronotherapy should be organised around host and/or parasite time is essential. Furthermore, the alignment between host and parasite rhythms itself may be a key determinant of treatment efficacy at different times of day.

**Table 1.** Previous research on the stage specificity of artemisinin derivatives in drug-sensitive parasites

	References
**Rings vs older IDC stages** ^a^	
No stage specificity observed	[12, 16–20]
Early IDC stages less sensitive than older IDC stages	[11, 17, 19, 21, 22–37]
Early IDC stages more sensitive than older IDC stages^b^	[16, 20, 33, 34, 37–44]
**Early vs late rings**	
Early rings more sensitive than late rings	[16, 27, 32, 33]
Early rings similarly sensitive as late rings	[42, 45]
Early rings less sensitive than late rings	[34]

a Older IDC stages include trophozoites and/or schizonts.

^b^ Many of these studies use the schizont maturation test (incubation of parasites with drugs until a certain percentage of parasites in control wells matures into schizonts), in which the duration of drug treatment is longer for the early IDC stages and thus is confounded with stage specificity.

In contrast to many other antimalarial drugs, artemisinins also target the early intra-erythrocytic developmental cycle (IDC) stages (‘rings’) of *Plasmodium* parasites, but studies report inconsistent results regarding the relative sensitivity of rings versus older IDC stages.

Understanding the relative contributions of host and parasite rhythms to antimalarial efficacy could resolve the disconnect between *in vitro* efficacy tests and *in vivo* treatment outcomes, reveal how temporary arrest (quiescence) at certain IDC stages confers tolerance to antimalarials and allow malaria treatment to be optimised by chronotherapy. Here, we use the rodent malaria parasite *Plasmodium chabaudi* to investigate whether both host and parasite rhythms contribute to antimalarial efficacy. We used the short-acting drug artemisinin to test for proof-of-principle, whilst acknowledging the ongoing importance of artemisinin-based therapies in malaria management. By manipulating whether IDC rhythms are aligned with host rhythms or not, and treating infections at different times of day with artemisinin, we tested whether drug efficacy depends on the parasite’s IDC stage, host time-of-day or their interaction. Additionally, we assessed putative correlates (haem and blood glucose concentration) of host rhythms and drug efficacy. Haem is an essential molecule for IDC progression, which parasites can biosynthesise, or scavenge from host haemoglobin [46]. Intracellular haem activates artemisinin [21, 47], and both haemoglobin concentration [4] and the rate of haem synthesis in red blood cells (RBCs) are under circadian control [48]. Thus, daily variation in haem levels could affect pharmacokinetics, with the expectation that artemisinin efficacy is boosted when haem levels are high. Blood glucose concentration also oscillates daily [49] and is a vital resource for parasites [50], suggesting parasites may be more sensitive to drugs when glucose concentration is low.

We reveal that parasites early in their IDC (rings) are less sensitive to artemisinin than mid-IDC parasites (trophozoites). Host time-of-day did not directly affect drug efficacy but the coordination between parasite and host rhythms did. Specifically, when the timing of the IDC was misaligned (mismatched) with host rhythms, rings exhibited even lower drug sensitivity and trophozoites became more sensitive. Whereas glucose levels did not correlate with drug efficacy, haem concentration at the point of treatment correlated positively with drug efficacy, but only in matched infections.

## METHODOLOGY

We carried out two experiments to test whether host and parasite rhythms affect artemisinin efficacy. The first experiment revealed intra-erythrocytic developmental cycle (IDC)-stage-specific efficacy and suggested an impact of host time-of-day. The second experiment tested whether the apparent role of host time-of-day was better explained by the alignment between parasite and host rhythms.

### Parasites and hosts

**Figure 1.**
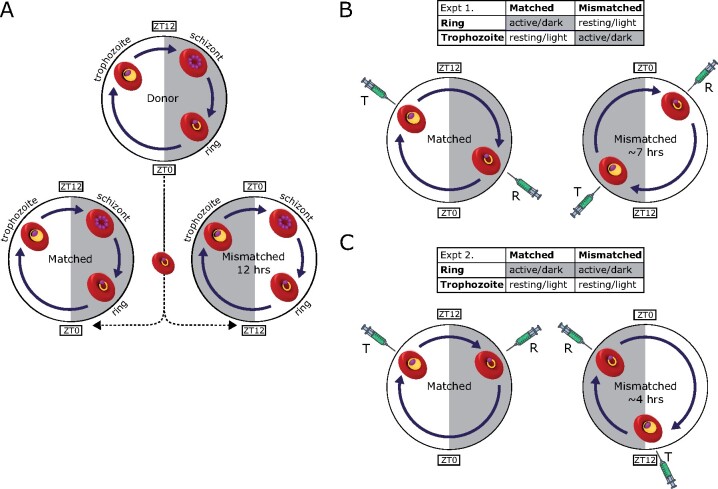
Experimental designs. Ring stage parasites were transferred from donor mice into recipient mice housed in the same photoschedule (host and parasite rhythms aligned: ‘matched’) or recipients housed in the reverse photoschedule (host and parasite rhythms misaligned: ‘mismatched’). Approximate timings when parasite IDC stages are abundant in the blood are indicated by infected red blood cells. (**A**). Due to rescheduling of parasites to the rhythms of their new hosts, parasite stages at the time of drug administration were misaligned from their host’s schedule by ∼7 and ∼4 h in experiments 1 (**B**) and 2 (**C**), respectively. Syringes indicate the treatment time for rings (R) and trophozoites (T), grey shading indicates lights off (i.e. mouse active phase). Note: artemisinin is a rapidly acting drug, with an elimination half-life of ∼23 min in mice [15].

We used the synchronous species *P. chabaudi chabaudi* (genotype CW) originally isolated from *Grammomys poensis* (previously called *Thamnomys rutilans*). Hosts were 7- to 11-week-old male C57BL/6 mice which had access to food *ad libitum* and drinking water was supplemented with 0.05% para-aminobenzoic acid [51]. *Mus musculus* is a natural host for some rodent malaria species [52]. All mice were kept in photoschedules of 12 h light-12 h dark, although the time-of-day at which lights went on/off varied between treatment groups within each experiment (Fig. 1). Mice were acclimatised (entrained) to their respective photoschedules for at least two weeks before infections. All procedures occurred in accordance with the UK Home Office regulations (Animals Scientific Procedures Act 1986; project licence number 70/8546) and were approved by the University of Edinburgh.

### Experimental design

Both experiments followed the same general set-up. We expanded cryopreserved parasites through 2–3 passages in donor mice kept in a 12 h light–12 h dark photoschedule (lights on: 7 am, lights off: 7 pm). This ensured parasites’ IDC schedule was aligned with host rhythms. To initiate experimental infections, we diluted blood from donor mice in citrate saline (0.85% w/v NaCl, 1.5% w/v trisodium citrate dihydrate) and passaged parasites by intravenous injection of ring stage infected red blood cells (iRBCs) into two groups of experimental mice simultaneously. One group of experimental mice were in the same photoschedule as the donor mice (lights on: 7 am, lights off: 7 pm), so that parasite and host rhythms were aligned in experimental hosts (‘matched’ infections). The other group of experimental mice was in the opposite photoschedule (lights on: 7 pm, lights off: 7 am), such that parasites transferred from donor to experimental mice experienced an instantaneous 12-h shift in host time-of-day (causing misaligned, ‘mismatched’ infections) (Fig. 1A). To ensure all hosts were infected from the same pool of parasites, we initiated experimental infections at 7 am; when lights went on for recipients of matched infections (Zeitgeber Time 0, ZT0), and at lights off for hosts of mismatched infections (ZT12) (Fig. 1A). We split both matched and mismatched infections into two groups that received drug treatment when parasites were either rings or trophozoites (as determined by microscopy), creating four treatment groups (Fig. 1B–C). Stage distributions at the time of treatment did not differ between matched and mismatched infections (Supplementary Table S1). Treated infections received a sub-curative dose of 50 mg/kg artemisinin (Alfa Aesar) dissolved in 50 µl dimethyl sulphoxide (DMSO) and administered by intraperitoneal injection. The short elimination half-life of artemisinin (∼23 min) [15] ensured that only the intended IDC stage was exposed to treatment. Placebo-treated control mice received 50 µl DMSO intraperitoneally when parasites were rings. Mismatched parasites reschedule to coordinate with their host’s feeding rhythms within a few IDCs [49, 53, 54]. Therefore, to minimise the extent of rescheduling in mismatched parasites, we administered drug treatment early in the infection and initiated infections with high parasite doses to ensure densities were high enough for accurate quantification.

### Experiment 1

We initiated infections (*n* = 40) with 10^8^ ring stage iRBCs and drug treated on Day 2 post infection (p.i.). At this point, the IDC of mismatched parasites was misaligned to host rhythms by ∼7 h (Fig. 1B). Based on this schedule, we administered artemisinin or placebo during the period of ring stage development (ZT20 for *n* = 8 matched infections and *n* = 4 matched controls, and ZT3 for *n* = 8 mismatched infections and *n* = 4 mismatched controls), or during the trophozoite stage (ZT8 for *n* = 8 matched infections and ZT15 for *n* = 8 mismatched infections) (Fig. 1B). We took blood samples from the tail vein just prior to drug administration (*t*_0_) to quantify parasites and measure haem and glucose levels, and also quantified parasites after 24 h (*t*_24_). We quantified parasites from 5-µl blood samples using a semi-automatic Kingfisher Flex Magnetic Particle Processor and MagMAX™-96 DNA Multi-Sample Kit (Thermo Fisher Scientific) with slight modifications from the standard protocol 4413021DWblood [55] and enumerated parasite genomes using qPCR targeting the CG2 gene (PCHAS_0620900, previously named PC302249.00.0) [56]. We measured blood glucose concentration using an Accu-Check^®^ Performa Nano device (Roche). For the haem assay (Heme Assay Kit, Sigma-Aldrich), absorbance was measured at 405 nm using a Multiskan Ascent 96/384 Plate Reader (MTX Lab Systems) in a single plate, following manufacturer’s instructions. We were unable to measure one haem sample (rings, matched infection).

### Experiment 2

The aim of the first experiment was to target rings and trophozoites in both matched and mismatched infections. This resulted in parasites in matched and mismatched infections being treated at times coupled to their hosts’ active or resting phase (Fig. 1B). Therefore, a difference in the sensitivity of rings in matched versus mismatched infections could be driven simply by physiological differences between matched and mismatched parasites [57], and/or by the effects of treating hosts in their active versus resting phase. Thus, Experiment 2 was designed such that we treated each IDC stage in the same host phase for both matched and mismatched infections. To achieve this, we initiated infections (*n* = 30) at a 10-fold lower parasite density (10^7^ ring stage iRBCs) than Experiment 1, followed by drug treatment on Day 3 p.i. This allowed mismatched parasites an additional IDC to reschedule, so that parasite and host rhythms in mismatched infections were only misaligned by ∼4 h at *t*_0_. Therefore, each IDC stage could be treated in the same host phase across matched and mismatched infections (Fig. 1C). Specifically, rings were treated in the active (dark) phase (ZT16 for *n* = 5 matched infections and *n* = 5 matched controls, and ZT20 for *n* = 5 mismatched infections and *n* = 5 mismatched controls), and trophozoites were treated in the resting (light) phase (ZT8 for *n* = 5 matched infections and ZT11.5 for *n* = 5 mismatched infections) (Fig. 1C). We took blood samples from the tail vein at *t*_0_ and *t*_24_. In contrast to Experiment 1, parasite densities were high enough for accurate quantification by microscopy. Thus, we calculated parasite densities by multiplying RBC densities as measured by flow cytometry (Beckman–Coulter counter) and determined the proportion of iRBCs by microscopic counting from thin blood smears stained with 20% Giemsa.

### Statistical analysis

Data were analysed with R v.3.6.3 (R Foundation for Statistical Computing, Vienna, Austria, https://www.R-project.org/) using the base R system unless otherwise indicated. To assess any differences between treatment groups at *t*_0_, we compared parasite densities, as well as glucose and haem levels for Experiment 1, between treatment groups using linear models with IDC ‘Stage’ (rings or trophozoites), ‘Alignment’ (matched or mismatched infections) and their interaction as explanatory variables. Parasite densities were log_10_-transformed to meet assumptions of normality and homogeneity of variance. Because the time-of-day of drug administration varied between groups, parasites in some groups had gone through an additional part-cycle of replication, resulting in differences in parasite densities at *t*_0_ (Supplementary Table S2 and Fig. S1). To correct for this, we analysed relative change (ΔParDens_rel_(*t*_0_, *t*_24_)) by fitting parasite density at *t*_24_ (ParDens(*t*_24_)), in linear models including an offset for parasite density at *t*_0_ (ParDens(*t*_0_)). Results are presented as the corresponding fold change:

$$\mathrm{relative}\;\mathrm{change}\;\mathrm{in}\;\mathrm{ParDens}\;\mathrm{post}\;\mathrm{treatment}\;(\Delta {\mathrm{ParDens}}_{\mathrm{rel}}\left(t_{0},t_{24}\right))=\frac{\mathrm{ParDens}(t_{24})}{\mathrm{ParDens}\;(t_{0})}$$

A relative change of 1 means that parasite densities were identical at *t*_0_ and *t*_24_, a decrease is indicated by <1, and an increase by >1. Note: values >1 in artemisinin-treated groups do not indicate that drugs were ineffective, merely that after drug treatment, parasites replicated to higher densities than they were at *t*_0_.

**Figure 2.**
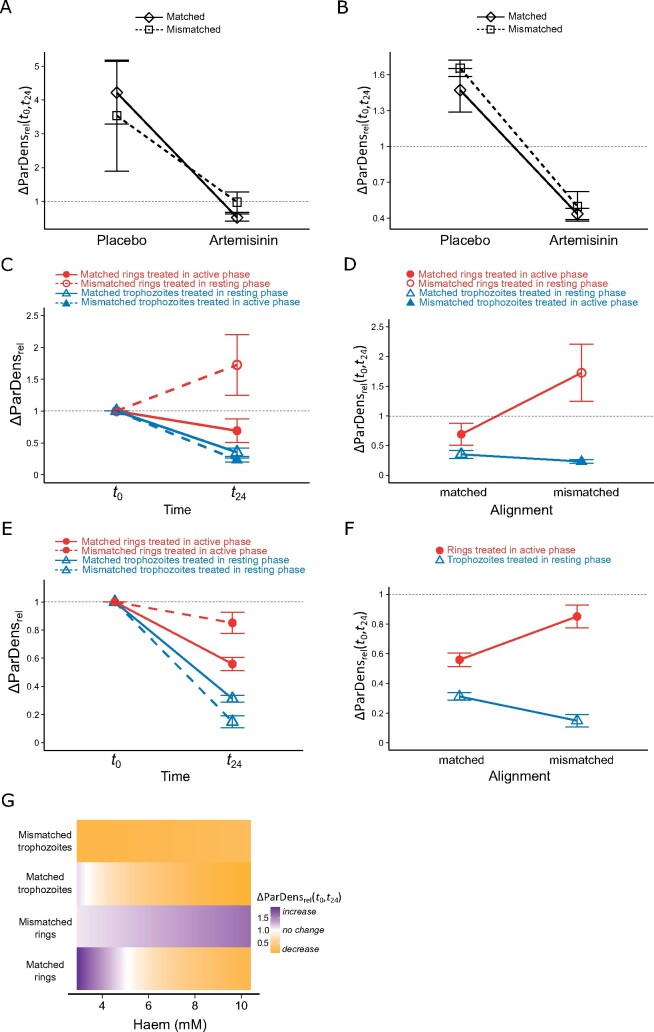
Effects of artemisinin drug treatment on parasite density. Relative change in parasite densities from 0 to 24 h post treatment (ΔParDens_rel_(*t*_0_, *t*_24_)) in Experiment 1 (**A**, **C**, **D**) and Experiment 2 (**B**, **E**, **F**). The horizontal dashed lines indicate no change in parasite density over time. Data represent the mean ± SEM. (**A**, **B**) Parasites were treated with placebo or artemisinin in matched (diamonds, solid lines) and mismatched infections (squares, dashed lines). All artemisinin-treated infections are combined regardless of which parasite stage was treated. (**C**-**F**) Parasites were treated as rings (red circles) or trophozoites (blue triangles), during the host’s active phase/dark (closed symbols) or resting phase/light (open symbols). Note: (**D**) and (**F**) are replotted versions of (**C**) and (**E**), respectively, to highlight the interaction between Alignment and Stage. (**G**) Heatmap indicating predicted ΔParDens_rel_(*t*_0_, *t*_24_) according to haem levels. Increasing parasite densities are shown in purple, decreasing parasite densities are shown in yellow, with white indicating no change.

To confirm artemisinin-treated infections had fewer parasites than placebo-treated controls regardless of whether infections were matched or mismatched, we tested a linear model including the terms ‘Drug’ (drug-treated or placebo-treated infections), ‘Alignment’ (matched or mismatched infections) and their interaction (Supplementary Table S3, Fig. 2A–B). To test our hypothesis that drug efficacy depends on IDC stage and alignment of host and parasite rhythms, linear models included the terms ‘Stage’ (rings or trophozoites), ‘Alignment’ (matched or mismatched infections) and their interaction as explanatory variables (plus haem or glucose levels for Experiment 1). For Experiment 1, we also constructed models using the term ‘Phase’ (host active or resting phase) instead of ‘Alignment’ (matched or mismatched infections) to test whether alignment or host phase best explained drug efficacy. We minimised nested models using maximum likelihood deletion tests, for which we report test statistics and *P*-values. We compared non-nested models by Akaike Information Criteria (corrected) (AICc) for small sample sizes; ‘MuMIn’ package). We report results for the minimally adequate models in the main text, and full statistical outcomes in Tables 2–3 and Supplementary Tables S2–S5. We present predictions of drug efficacy according to haem levels at *t*_0_ based on the minimised model shown in Table 2B with a variation in haem concentrations ranging from 2.9 to 10.4 mM in 0.01 steps, and parasite density at the time of treatment set to the mean for each of the four treatment groups.

**Table 2.** Analyses of how drug efficacy depends on parasite intra-erythrocytic developmental cycle (IDC) stage, the alignment of parasite and host rhythms, and putative physiological correlates

A—Drug efficacy varies with parasite IDC stage and the alignment of parasite and host rhythms		
Expt. 1: log_10_ ParDens *t*_24_∼Stage×Alignment + offset (log_10_ ParDens *t*_0_)		
**Stage×Alignment**	***F*_(1,28)_ = 10.19**	***P *=* *0.003**
Expt. 2: log_10_ ParDens *t*_24_∼Stage×Alignment + offset (log_10_ ParDens *t*_0_)		
**Stage×Alignment**	***F*_(1,16)_ = 13.52**	***P *=* *0.002**

B—Haem levels positively correlate with drug efficacy in matched infections		
Expt. 1: log_10_ ParDens *t*_24_∼Stage×Alignment×Haem + offset (log_10_ ParDens *t*_0_)		
Stage×Alignment×Haem	*F*_(1,23)_ = 0.06	*P *=* *0.809
Stage×Haem	*F*_(1,24)_ = 0.22	*P *=* *0.641
**Alignment×Haem**	***F*_(1,25)_ = 6.24**	***P *=* *0.019**
**Stage×Alignment**	***F*_(1,25)_ = 10.73**	***P *=* *0.003**

C—Glucose levels do not correlate with drug efficacy		
Expt. 1: log_10_ ParDens *t*_24_∼Stage×Alignment×Glucose + offset (log_10_ ParDens *t*_0_)		
Stage×Alignment×Glucose	*F*_(1,24)_ = 0.61	*P *=* *0.441
Stage×Glucose	*F*_(1,25)_ = 0.73	*P *=* *0.402
Alignment×Glucose	*F*_(1,26)_ = 0.49	*P *=* *0.488
Glucose	*F*_(1,27)_ = 1.99	*P *=* *0.170
**Stage×Alignment**	***F*_(1,28)_ = 10.19**	***P *=* *0.003**

Results of full linear statistical models including parasite densities (ParDens) at the time of drug administration (*t*_0_) and 24 h later (*t*_24_) are reported. Terms included in the final models are in bold.

**Table 3.** Analyses of how drug efficacy depends on parasite intra-erythrocytic developmental cycle (IDC) stage, host phase and putative physiological correlates

A—Drug efficacy varies with parasite IDC stage and host phase at drug administration		
Expt 1: log_10_ ParDens *t*_24_∼Stage×Phase + offset (log_10_ ParDens *t*_0_)		
	ΔAICc = 1.12	
Stage×Phase	*F*_(1,28)_ = 1.53	*P *=* *0.226
**Phase**	***F*_(1,29)_ = 10.01**	***P *=* *0.004**
**Stage**	***F*_(1,29)_ = 38.11**	***P *<* *0.001**

B—Haem levels positively correlate with drug efficacy in matched infections		
Expt 1: log_10_ ParDens *t*_24_∼Stage×Phase×Haem + offset (log_10_ ParDens *t*_0_)		
	ΔAICc = 7.33^a^	
**Stage×Phase×Haem**	***F*_(1,23)_ = 5.87**	***P *=* *0.024**

C—Glucose levels do not correlate with drug efficacy		
Expt 1: log_10_ ParDens *t*_24_∼Stage×Phase×Glucose + offset (log_10_ ParDens *t*_0_)		
	ΔAICc = 1.12	
Stage×Phase×Glucose	*F*_(1,24)_ = 0.24	*P *=* *0.628
Stage×Glucose	*F*_(1,25)_ = 0.50	*P *=* *0.485
Stage x Phase	*F*_(1,26)_ = 0.91	*P *=* *0.349
Phase x Glucose	*F*_(1,27)_ = 2.93	*P *=* *0.098
Glucose	*F*_(1,28)_ = 0.85	*P *=* *0.365
**Phase**	***F*_(1,29)_ = 10.01**	***P *=* *0.004**
**Stage**	***F*_(1,29)_ = 38.11**	***P *<* *0.001**

a Model with Alignment (Table 2A–C) fits better than model with Phase (Table 3A–C).

Results of full linear statistical models including parasite densities (ParDens) at the time of drug administration (*t*_0_) and 24 h later (*t*_24_) are reported. Models presented in Table 3 correspond to models in Table 2, with the term ‘Alignment’ replaced by the term ‘Phase’. Terms included in final models are in bold. ΔAICc: delta Akaike Information Criteria (corrected) compared to the associated model using ‘Alignment’ in Table 2. ΔAICc > 2 indicates that models are different and the better fitting model (‘Alignment’ or ‘Phase’) is indicated.

## RESULTS

### Assumptions of the experimental design

Parasites were treated at either ring or trophozoite stage and, as expected, the intended intra-erythrocytic developmental cycle (IDC) stage predominated in the blood in all treatment groups just prior to treatment (*t*_0_) (Supplementary Table S1). Because we administered treatment to specific IDC stages, parasite densities at *t*_0_ differed between treatment groups (Experiment 1, Stage × Alignment interaction: *F*_(1,28)_ = 5.48, *P *=* *0.027; Experiment 2, Stage × Alignment interaction: *F*_(1,17)_ = 1.90, *P *=* *0.187, Stage: *F*_(1,18)_ = 11.14, *P *=* *0.004) (Supplementary Table S2 and Fig. S1). This confirms the need to measure drug efficacy as the relative change in parasite density from 0 to 24 h post artemisinin treatment (ΔParDens_rel_(*t*_0_, *t*_24_)).

In both experiments, artemisinin-treated infections had lower relative parasite densities than placebo-treated controls (Experiment 1, Drug: *F*_(1,38)_ = 29.94, *P *<* *0.001; Experiment 2, Drug: *F*_(1,28)_ = 28.64, *P *<* *0.001) (Supplementary Table S3). Specifically, parasite densities increased in placebo-treated infections (mean ΔParDens_rel_(*t*_0_, *t*_24_) ± SEM 4.22 ± 0.93 for matched and 3.54 ± 1.64 for mismatched infections in Experiment 1, and 1.47 ± 0.18 for matched and 1.65 ± 0.07 for mismatched infections in Experiment 2). In contrast, parasite densities decreased or remained unchanged in artemisinin-treated infections (mean ΔParDens_rel_(*t*_0_, *t*_24_) ± SEM 0.52 ± 0.10 for matched and 0.98 ± 0.30 for mismatched infections in Experiment 1, and 0.44 ± 0.05 for matched and 0.50 ± 0.12 for mismatched infections in Experiment 2) (Fig. 2A–B).

### Impact of host and parasite rhythms on drug efficacy

Drug efficacy (ΔParDens_rel_(*t*_0_, *t*_24_)) in Experiment 1 varied significantly according to whether we treated rings or trophozoites and whether host and parasite rhythms were aligned (Stage×Alignment interaction: *F*_(1,28)_ = 10.19, *P *=* *0.003) (Table 2A). In matched infections, rings were less sensitive to artemisinin than trophozoites (mean ΔParDens_rel_(*t*_0_, *t*_24_) ± SEM 0.69 ± 0.18 for matched rings and 0.35 ± 0.07 for matched trophozoites). When parasite and host rhythms were mismatched, drug efficacy was enhanced for trophozoites and weakened for rings (mean ΔParDens_rel_(*t*_0_, *t*_24_) ± SEM 1.73 ± 0.48 for mismatched rings and 0.23 ± 0.03 for mismatched trophozoites) (Fig. 2C–D). Thus, misalignment between parasite and host rhythms exacerbated the stage specificity of artemisinin.

### Separating the effects of rhythm alignment and host phase

In Experiment 1, alignment co-varied with treatment during the host’s active (dark) or resting (light) phase because upon misalignment, the appearance of each IDC stage in the blood also shifted to the opposite host phase compared to aligned infections (Fig. 1B). Therefore, we sought to determine whether the observed differences in drug efficacy in matched and mismatched infections were better explained by alignment or by host phase at the time of treatment. Statistical models in which we replaced ‘Alignment’ with the administration of artemisinin during the host’s active or resting phase (‘Phase’) explained drug efficacy equally well (ΔAICc < 2, Table 3A). Rings were less sensitive to artemisinin than trophozoites, and treatment during the host’s active phase (dark) was more effective than in the host’s resting phase (light) (Stage×Phase interaction: *F*_(1,28)_ = 1.53, *P *=* *0.226, Phase: *F*_(1,29)_ = 10.01, *P *=* *0.004, Stage: *F*_(1,29)_ = 38.11, *P* < 0.001) (Table 3A). Hence, the differences in stage specificity between matched and mismatched infections could have equally resulted from increased artemisinin efficacy when administered during the host’s active phase as from misalignment between hosts and parasites. We employed a second experiment to differentiate between these two scenarios.

Administering treatment 1 day later in Experiment 2 enabled parasites to reschedule further. Consequently, rings in Experiment 2 could be treated during the host’s active phase for both matched and mismatched infections, whilst all trophozoites were treated during the resting phase (Fig. 1C). If the effects of alignment between host and parasite rhythms observed in Experiment 1 are simply due to the effects of different host phases on drug action, there should have been no difference in sensitivity within each IDC stage in Experiment 2, as they were now each treated in the same host phase. Instead, efficacy in Experiment 2 did vary significantly according to both IDC stage and the alignment of parasite and host rhythms (Stage×Alignment interaction *F*_(1,16)_ = 13.52, *P *=* *0.002) (Table 2A, Fig. 2E–F), revealing alignment as an important driver. Analogous to Experiment 1, rings in matched infections were less sensitive than trophozoites (mean ΔParDens_rel_(*t*_0_, *t*_24_) ± SEM 0.56 ± 0.05 for matched rings and 0.31 ± 0.03 for matched trophozoites), and this difference was exacerbated by mismatch (mean ΔParDens_rel_(*t*_0_, *t*_24_) ± SEM 0.85 ± 0.08 for mismatched rings and 0.15 ± 0.04 for mismatched trophozoites). Therefore, alignment between host and parasite rhythms has a greater influence on IDC-stage-specific drug efficacy than host phase *per se*.

### Physiological correlates of stage-specific drug sensitivity

Haem concentrations at *t*_0_ in Experiment 1 ranged from 2.95 to 10.38 mM, but did not differ significantly between treatment groups (Stage×Alignment interaction: *F*_(1,27)_ = 1.30, *P *=* *0.264, Alignment: *F*_(1,28)_ = 0.01, *P *=* *0.941, Stage: *F*_(1,29)_ = 0.45, *P *=* *0.508) (Supplementary Table S4). Despite a lack of significant between-group variation in haem levels, the statistical model explaining drug efficacy by ‘Stage’ and ‘Alignment’ (Table 2A) was significantly improved by the interaction between ‘Alignment’ and ‘Haem’ (Alignment×Haem interaction: *F*_(1,25)_ = 6.24, *P *=* *0.019) (Table 2B). Including haem levels at *t*_0_ in these analyses explained 5% more of the variance in ΔParDens_rel_(*t*_0_, *t*_24_). Haem concentrations correlated positively with artemisinin efficacy for both rings and trophozoites, but only in matched infections (Fig. 2G). When using ‘Phase’ in the analyses, ‘Haem’ also added a significant improvement to the models (Stage×Phase×Haem interaction: *F*_(1,23)_ = 5.87 *P *=* *0.024) (Table 3B). However, this model did not explain drug efficacy as well as the model including ‘Stage’, ‘Alignment’ and ‘Haem’ (ΔAICc = 7.33, Table 3B). This supports our finding that alignment is a more important driver of IDC-stage-specific drug efficacy than host phase.

Blood glucose concentrations at *t*_0_ in Experiment 1 ranged from 7.7 to 12.4 mmol/L. The alignment of parasite and host rhythms did not affect blood glucose concentrations (Stage×Alignment interaction: *F*_(1,28)_ = 0.14, *P *=* *0.709; Alignment: *F*_(1,29)_ = 3.10, *P *=* *0.089), but blood glucose was 9.6% higher when trophozoites were treated (mean concentration = 10.77 ± 0.23 mmol/L) compared to when rings were treated (mean concentration = 9.83 ± 0.36 mmol/L) (Stage: *F*_(1,30)_ = 5.00, *P *=* *0.033) (Supplementary Table S5). However, blood glucose levels did not explain any additional variance in ΔParDens_rel_(*t*_0_, *t*_24_) of infections treated as rings or trophozoites, irrespective of using ‘Alignment’ or ‘Phase’ in the analyses (Tables 2C and 3C), revealing that drug efficacy was unaffected by blood glucose concentrations at *t*_0_.

## DISCUSSION

We experimentally separated parasite intra-erythrocytic developmental cycle (IDC) rhythms and host rhythms *in vivo* and used a fast-acting antimalarial to test their independent and interacting effects on drug efficacy. Our experiments revealed three phenomena. First, mid-ring stages of *P. chabaudi* are less sensitive to artemisinin than mid-trophozoites. Second, alignment between parasite and host rhythms differentially affects drug sensitivity of rings and trophozoites; mismatch causes rings to become less sensitive and trophozoites to become more sensitive. Third, unlike blood glucose concentrations, haem levels in the blood at the time of drug administration (*t*_0_) positively correlate with artemisinin efficacy (regardless of the IDC stage treated) but only in infections where host and parasite rhythms are aligned.

That we find rings are less sensitive to artemisinin treatment than trophozoites, is in keeping with other studies that also challenge mid-rings, rather than hypersensitive very early rings [16, 27, 32, 33] (Table 1). There are several non-mutually exclusive explanations for the lower sensitivity of mid-rings. First, low levels of haemoglobin digestion early in the IDC might cause rings to be exposed to lower levels of haem-activated artemisinin compared to trophozoites [21, 33]. Although we were unable to detect variation in haem levels between IDC stages, this was potentially due to high between-host variation. Repeated measures of haem concentration from each infection throughout an IDC might provide a more sensitive test of this hypothesis. Second, rings may be better able to survive exposure to activated artemisinins than other IDC stages (e.g. [33, 58]). For example, lower levels of ubiquitination and haemoglobin digestion [20, 21] suggest rings may have an under-used proteasome and redox buffering capacity, which they could harness when faced with artemisinin-induced homeostatic imbalance [59]. Furthermore, drug-induced quiescence generally occurs at ring stage [20, 26, 27, 60], and various artemisinin-resistant mutations confer enhanced protection for rings (e.g. [61]). Perhaps IDC arrest at ring stage has evolved as a survival strategy because the robust nature of rings means this stage is most likely to withstand varied environmental insults.

The differential impact of the alignment between IDC stages and host rhythms on drug sensitivity of rings versus trophozoites cannot simply be explained by the effects of the host’s active versus resting phase on drug activity. When the alignment between parasite and host rhythms was lost, regardless of whether treatment was administered during the host’s active or resting phase, trophozoites became more sensitive and rings less sensitive to artemisinin treatment, thus aggravating the stage-specific differences in drug efficacy. Artemisinin is an important currently used antimalarial, and if our findings translate to human malaria infections, then timing administration of this short-acting drug against trophozoites would maximise efficacy. The World Health Organisation recommends artemisinin combination therapy to be administered for 3 days against the human malaria parasite *P. falciparum* [62, 63], which has a 48-h IDC (reviewed in [10]). Whilst we would not advocate withholding treatment from ill patients, it might be beneficial to ensure 2 of the 3 days are timed to target trophozoites. If novel drugs can be developed to disrupt the alignment between the IDC schedule and host rhythms (e.g. by modulating nutrient sensing by the parasite [64]), then administering such drugs alongside the first dose of traditional antimalarials might render subsequent doses targeting trophozoites, more effective.

Finding targets for IDC-schedule-disrupting drugs will be facilitated by understanding why mismatch exacerbates stage-specific differences in drug sensitivity. Clues could lie in the altered gene expression patterns recently uncovered in mismatched *P. chabaudi* parasites [57]. This includes genes involved in DNA replication, oxidation-reduction processes, ubiquitin-mediated proteolysis and proteasome pathways and energy metabolism [57]. Many of these pathways are also linked to artemisinin resistance, for example: (i) proteasome inhibitors synergise artemisinin efficacy [20, 65]; (ii) a delayed progression through the IDC protects parasites from various drugs including artemisinins [20, 24, 26, 27, 40, 60]; (iii) nutrient limitation can prolong the maturation period of rings *in vitro* [66, 67], whilst disproportionally affecting artemisinin-tolerant parasites that rely more on exogenous amino acids to mature from rings to trophozoites [68]; and (iv) transcriptional profiles of *P. falciparum* lines selected for artemisinin resistance show transcriptional changes for similar pathways [23, 69].

Although studies of chronotherapy against infections are scarce, diurnal changes in drug efficacy have been observed for both fungal and bacterial infections. Treatment of *Candida albicans* was most effective in the resting phase of mice [70], whereas *E. coli* in rats was best killed during the active phase [71]. In both studies, the impact of timing faded when higher drug doses were used, perhaps indicating the importance of chronotherapy in cases where drug doses are constrained due to toxicity or when pathogens are not completely cleared by drugs—for example when combatting partially resistant populations. When seemingly adequately applied antimalarials do not immediately eliminate all parasites [72], sublethal drug doses may minimise selection for resistance [73] and virulence [74]. In addition, such doses may maximise the impact of chronotherapy on parasite clearance alongside its potential benefits in reducing drug toxicity [75].

Precise timing of drug administration could also affect efficacy via downstream effects on the immune system. In a murine bone injury model, drug administration during the active period promoted anti-inflammatory cytokines over a pro-inflammatory response, and in turn resulted in improved healing, compared to treatment during the resting period [76]. By the same token, chronotherapy for COVID-19 has been suggested, with the aim to attenuate the typically detrimental inflammatory cascade in patients in the afternoon and evening, without interfering with the beneficial inflammatory response during the day [77]. Artemisinin derivatives attenuate pro-inflammatory immune responses (reviewed in [78]), which could be harnessed to mitigate the pathology associated with schizogony-induced inflammation (reviewed in [79]). Treating mid-to-late trophozoites could thus be doubly helpful: parasites would be targeted at their most vulnerable IDC stage, and this occurring shortly before schizogony could maximise artemisinin’s immunosuppressive properties against the impeding cytokine storm caused by any surviving parasites.

## CONCLUSIONS AND IMPLICATIONS

Our experiments were performed *in vivo* using a nocturnal host and the rodent malaria parasite *P. chabaudi*, which has a 24-h intra-erythrocytic developmental cycle (IDC). Care should be taken when extrapolating results to human malaria, among others because *P. falciparum* has a 48-h IDC and humans are not nocturnal. However, like *P. chabaudi*, many genes of *P. falciparum* are transcribed with 24-h periodicity [57], including genes involved in processes that lose rhythmicity in misaligned infections in *P. chabaudi*, and have been implicated in artemisinin resistance in both rodent malarias and *P. falciparum* (e.g. carbohydrate metabolism, DNA replication and the ubiquitin proteasome system [23, 57, 69]). Furthermore, like mice, humans also have active and resting phases, and misalignment between host and *P. falciparum* IDC rhythms can occur, with infections even becoming asynchronous. When uncomplicated malaria cases are diagnosed, information on host internal time as well as parasite IDC stage distribution and synchronicity could be collected by microscopy [80] or qPCR [81] to infer the timing of the IDC rhythm and determine when parasites are best targeted. The impact of chronotherapy should be most pronounced in highly synchronous infections, when multi-day treatment regimens repeatedly target the same IDC stage in subsequent replication cycles [13, 82], and when parasite and host rhythms are naturally or forcefully misaligned. Finally, if timed treatment is not achievable, then extending artemisinin combination therapy to include not 3 (1.5 IDCs) but 4 days (2 IDCs) would make treatment efficacy less dependent on the sensitivity of the parasite IDC stage that happens to predominate at the start of treatment [13, 20, 58, 82].

## Supplementary Material

eoab013_Supplementary_Data
