# Supplementary material for: Daily rhythms of both host and parasite affect antimalarial drug efficacy
Source: Evol Med Public Health. 2021 Apr 26;9(1):208–19. doi: 10.1093/emph/eoab013 (PMC8284615; doi:10.1093/emph/eoab013)
Supplement: eoab013_Supplementary_Data [file eoab013_supplementary_data.docx]

**Supplementary Information**

**Supplementary Tables**

**Supplementary Table S1. Parasite stage structure at the time of drug administration (*t*_0_).** In both experiments, rings and trophozoites were drug treated in matched and mismatched infections. In all treatment groups, the expected intra-erythrocytic developmental cycle (IDC) stage predominated in the blood at *t*_0_. The stage distributions of matched and mismatched infections at *t*_0_ did not differ significantly in terms of stage % for either Experiment 1 (Stage×Alignment interaction: *F*_(1,28)_=2.19, *p*=0.150, Alignment: *F*_(1,29)_=0.02, *p*=0.886) or Experiment 2 (Stage×Alignment interaction: *F*_(1,16)_=0.70, *p*=0.415, Alignment: *F*_(1,17)_=0.05, *p*=0.824), and the SEM of stage percentages were also very similar. Note: that trophozoites generally constitute a lower proportion of stages compared to rings at *t*_0_, is likely due to sequestration beginning at the trophozoite stage.

| Experimental group | Experiment 1 | Experiment 2 |
| --- | --- | --- |
| Matched rings | 90%±1.01 rings | 83%±2.29 rings |
| Mismatched rings | 87%±1.83 rings | 86%±2.92 rings |
| Matched trophozoites | 73%±2.03 trophozoites | 71%±2.45 trophozoites |
| Mismatched trophozoites | 75%±1.72 trophozoites | 70%±2.06 trophozoites |

**Supplementary Table S2. Parasite densities at the time of drug administration vary between experimental groups.** Results of full linear statistical models including parasite densities (ParDens) at the time of drug administration (*t*_0_) and 24 hrs later (*t*_24_), are reported. Terms included in the final models are in bold.

| Expt. 1: log_10_ ParDens *t*_0_~Alignment×Stage | | | | |
| --- | --- | --- | --- | --- |
| Stage×Alignment | ***F*_(1,28)_ = 5.48** |  | ***p* = 0.027** | |
| Expt. 2: log_10_ ParDens *t*_0_~Alignment×Stage | | | |  |
| Stage×Alignment | *F*_(1,16)_ = 1.90 | | *p* = 0.187 |  |
| Alignment | *F*_(1,17)_ = 0.43 | | *p* = 0.522 |  |
| Stage | ***F*_(1,18)_ = 11.14** | | ***p* = 0.004** |  |

**Supplementary Table S3. Artemisinin reduces parasite densities compared to placebo.** Results of full linear statistical models including parasite densities (ParDens) at the time of drug administration (*t*_0_) and 24 hrs later (*t*_24_), are reported. Terms included in the final models are in bold.

| Expt. 1: log_10_ ParDens *t*_24_~Drug×Alignment + offset (log_10_ ParDens *t*_0_) | | |  |  |
| --- | --- | --- | --- | --- |
| Drug×Alignment | *F*_(1,36)_ = 0.86 | *p* = 0.359 | | |
| Alignment | *F*_(1,37)_ = 0.17 | *p* = 0.682 | | |
| Drug | ***F*_(1,38)_ = 29.94** | ***p* < 0.001** | | |
| Expt. 2: log_10_ ParDens *t*_24_~Drug×Alignment + offset (log_10_ ParDens *t*_0_) | | | |  |
| Drug×Alignment | *F*_(1,26)_ = 0.53 | *p* = 0.473 | | |
| Alignment | *F*_(1,27)_ = 0.22 | *p* = 0.640 | | |
| Drug | ***F*_(1,28)_ = 28.64** | ***p* < 0.001** | | |

**Supplementary Table S4.** **Haem levels at the time of drug administration in Experiment 1 do not vary between experimental groups.** Results of the full linear statistical model are reported. Terms included in the final model are in bold.

| Haem~Alignment×Stage | | |
| --- | --- | --- |
| Stage×Alignment | *F*_(1,27)_ = 1.30 | *p* = 0.264 |
| Stage | *F*_(1,29)_ = 0.45 | *p* = 0.508 |
| Alignment | *F*_(1,28)_ = 0.01 | *p* = 0.941 |

**Supplementary Table S5.** **Blood glucose levels at the time of drug administration in Experiment 1 vary with parasite intra-erythrocytic developmental cycle (IDC) stage.** Results of the full linear statistical model are reported. Terms included in the final model are in bold.

| Glucose~Alignment×Stage | | |
| --- | --- | --- |
| Stage×Alignment | *F*_(1,28)_ = 0.14 | *p* = 0.709 |
| Alignment | *F*_(1,29)_ = 3.10 | *p* = 0.089 |
| Stage | ***F*_(1,30)_ = 5.00** | ***p* = 0.033** |

**Supplementary Figure**


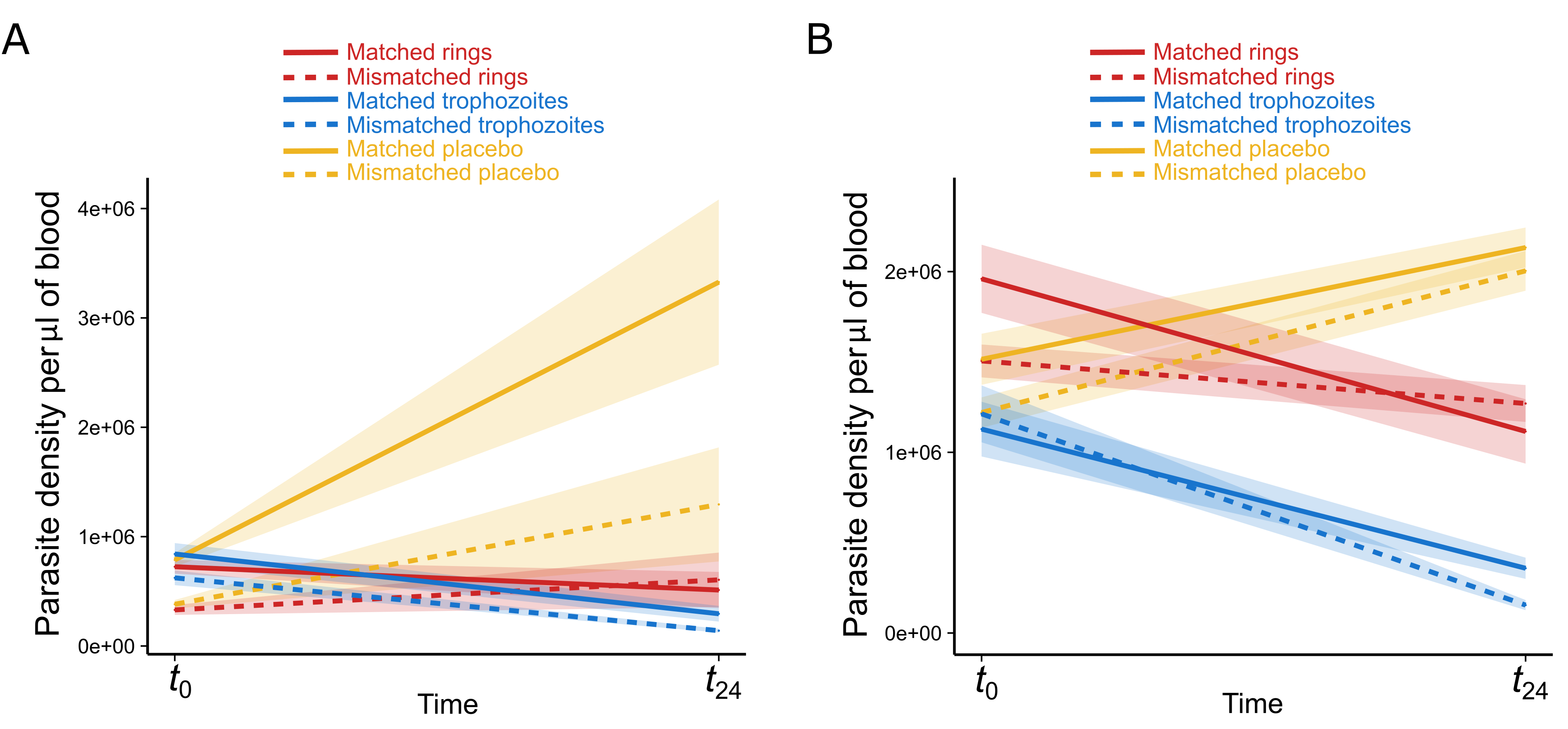


**Supplementary Figure S1.** **Artemisinin treatment reduces parasite densities compared to placebo.** Parasite densities 0-24 hrs after the administration of artemisinin or placebo in matched infections (solid lines) or mismatched infections (dashed lines) in Experiment 1 (**A**) and Experiment 2 (**B**). Parasites were either treated at ring stage (red) or at trophozoite stage (blue). Control hosts received placebo at ring stage (yellow). All artemisinin-treated groups experienced restricted parasite growth compared to parasites in control groups. Data represent the mean+s.e.m.
